# Advancements and challenges in methodological approaches for game-based health interventions: a scoping review

**DOI:** 10.3389/fdgth.2025.1561422

**Published:** 2025-03-24

**Authors:** Shaina Glass, Alexia Galati

**Affiliations:** ^1^ Department of Psychological Science, University of North Carolina at Charlotte, Charlotte, NC, United States; ^2^ Health Psychology Ph.D. Program, University of North Carolina at Charlotte, Charlotte, NC, United States

**Keywords:** gamification, health interventions, game-based approaches, serious games, Applied Game Design

## Abstract

**Introduction:**

Applying game design techniques to create engaging health interventions has become more common, though still met with challenges and criticisms. This scoping literature review evaluates the extent to which recent health-based game intervention studies have improved from past criticisms around the process of game development, theoretical grounding, and implementation in terms of research design.

**Methods:**

Following a search of relevant databases and an AI tool (Elicit.org), 26 published articles met our selection criteria of reporting a game-based health intervention task developed by the article's authors. In each article, the reported theoretical grounding, use of game mechanics, and methodologies for developing and implementing game-based interventions were assessed. Our procedure involved coding for psychological or game design theories, game mechanics, and the research methods and design approaches used for intervention development. We reasoned that articles grounded in theory would be more likely to report effective methodologies and support for their design choices.

**Results:**

Our findings revealed that authors frequently used quantitative methods to determine intervention impact, explicitly referenced psychological (vs. game design) theory more frequently, and used more than one game mechanic in the interventions. In line with recommendations, the majority of studies used large sample sizes and applied their interventions in real-world settings.

**Discussion:**

Despite these improvements, we identified areas of growth: utilizing interdisciplinary teams, user-centered and iterative approaches, and standardizing the reporting of intervention design components. This review is intended to inform the future of applied game design in health contexts.

## 1 Introduction

Applied Game Design, the method of creating games for non-gaming contexts, is on the rise in cognitive and healthcare settings (1). This method has promise for health behavior change: the observation of positive changes in behavior that lead to better health outcomes (2). Research findings about the effectiveness of Applied Game Design in health contexts have been mixed, leading researchers to debate whether it is an effective method for improving health outcomes. However, the misuse of Applied Game Design along with the use of weak study designs (3, 4) could contribute to these mixed results and obfuscate its effectiveness as a tool for delivering treatment. Given the increasing use and accessibility of game-based applications, it is important to continue evaluating the impact of Applied Game Design on the efficacy of health interventions.

In this paper, we take the position that Applied Game Design would be most beneficial when it is informed by design theory and by psychological findings and frameworks. With this starting point, after defining some key constructs relating to Applied Game Design, its use in behavioral interventions is discussed. Then, an overview of the use of Applied Game Design in health behavior change and critiques of the literature is provided. Next follows a review and evaluation of the extent to which theory and methods from game design and behavioral and health psychology have been used to inform the design of game-based health interventions. This evaluation allows us to establish whether recent research addresses prior critiques and to identify areas of growth for the development of future game-based interventions. Finally, recommendations for future directions utilizing Applied Game Design and their assessment are provided.

### 1.1 What is Applied Game Design?

Applied Game Design commonly refers to the creation of games and use of design techniques and methods for non-gaming contexts such as in health and education. Applied Game Design includes adding game mechanics and elements to tasks, such as story, aesthetics, and technology, not originally conceived as a game, and designing games specifically for non-gaming contexts (1, 5). Applied Game Design encompasses other common terms such as gamification and serious games (1, 5–7), along with applied games and game-based approaches (4).

In game design, a set of elements are used to form a game experience that leads to play and improved engagement. A game is thought to comprise four elements: technology, story, aesthetics, and mechanics (8). Technology refers to any material that makes the game possible, be it pen and paper or a computer. The story provides the reason for playing a game and gives background to the gameplay and environment. Aesthetics is the look and feel of a game. Lastly, mechanics encompasses the procedures and rules that make up a game. Mechanics can consist of objects, attributes (e.g., space, time), and states (e.g., the current state of the game or character) (8).

Mechanics are what dictate gameplay and are vital to eliciting player engagement and motivation. In the context of health-related applications, improved motivation and engagement are important in that they increase adherence to interventions, potentially contributing to positive health outcomes and behavior change (1, 9). The categorization of mechanics in the literature can vary. A common categorization of mechanics distinguishes between goals (providing progress through achievements, milestones, quests, and levels), status (bringing social influence through rankings or the sharing of achievements), randomness (implemented through events not impacted by user action to give the appearance of luck), appointment (engaging the user at set times), scoring (providing feedback to through points and bonuses), and immersion (emerging from the game through story, character roles, and the ability to explore) (1). However, other categorizations distinguish between critical, fatal, incentive, and avoidance mechanics (10), and performance, ecological, social, fictional, and personal mechanics (11). The specific typology notwithstanding, it is important to evaluate how mechanics contribute to the development of effective interventions.

### 1.2 Advantages of Applied Game Design

In Applied Game Design, game design elements and mechanics are used to enhance learning and encourage behavior change through improving engagement, motivation, and its potential for wide dissemination. The game elements described in the previous section, along with others, are thought to improve engagement in the task, including emotional involvement and passion (1, 5, 8). Game elements can place participants in a state of flow, a period of intense engagement, and immersion, a state of deep involvement with and focus on the task (1, 12). Game elements elicit enjoyment and positive emotions (5) by introducing challenge and fun while allowing players to achieve goals in a safe environment (13, 14). Enjoyment in a task is thought to contribute to better performance outcomes, including better retention in learning contexts, which is why applied game design is already being applied in various contexts, such as education (14).

Additionally, game elements can also have a positive impact on participants' motivation by providing intrinsic and extrinsic motivators (5). Intrinsic motivation refers to driving actions through internal satisfaction, such as curiosity and overcoming challenges (14). Extrinsic motivation refers to external drivers, such as rewards (14). Games are typically designed to be inherently challenging, leveraging goal setting theory, which posits that setting difficult but achievable goals motivates individuals (5, 15). Games can also improve motivation by providing clear and quick communication on performance (goals, scoring, rewards, etc.), which is associated with individuals feeling competent in their actions and satisfying needs for praise and accomplishment (5, 14, 16). Feedback during gameplay guides participants on whether their actions are right or wrong, and is associated with users reporting more positive experiences and spending more time on the task (5).

Games also have the unique property of being easily disseminated (12, 16). A significant draw for Applied Game Design is the increased reach it offers: this delivery method can be used to reach individuals that are not able to seek out medical or mental health care, overcoming the limitations of administering these tasks in a lab or a health care provider setting (12). An example of this can be seen in the Personal Zen game (37), a gamified version of an empirically tested attentional bias modification task to reduce anxiety. Participants are able to play this game on their own time on their personal smartphone, outside of the lab, and still experience the effects of reduced anxiety (12, 17). Game-based tasks, especially those accessible online, also support the feasibility of recruiting large and diverse samples of participants (15, 18). Due to the advantages of Applied Game Design, researchers across many fields in the health sciences, including clinical psychology, have leveraged its use in interventions.

### 1.3 Applied Game Design in health

Applied Game Design has been applied not only to educational contexts and cognitive training tasks, but also to health behavior change interventions targeting the improvement of physical health, mental health, and health behaviors (4, 12, 19–21). It has been used to encourage the use of healthy behaviors such as self-monitoring, better eating habits, physical activity, and sunscreen use, and to discourage unhealthy habits, such as substance abuse (4, 20, 21). For mental health, Applied Game Design has resulted in games in which participants practice skills from cognitive behavior therapy, cognitive training, and positive psychology with the goal of decreasing depressive symptoms (12). These tasks have been tested in the lab and outside the lab in both cross-sectional and longitudinal studies (4, 12, 20, 21).

Over the years, reviews have offered some insight into how game-based interventions are commonly designed. Some reviews have revealed that the mechanics implemented most frequently in game-based interventions are the use of levels [this is included in the “goal” category by (1)], points/scoring, rewards [these are included in the “scoring” category by (1)], and use of a narrative/story. The most common of these in game-based health interventions is the use of points/scoring (3, 22), which provide clear feedback to the player about their performance (1). The number of game mechanics used varies across tasks, with the majority of studies implementing around five game mechanics in their task (3).

The extant research still leaves open queries about the use of game mechanics, including which game mechanics are best for improving motivation and engagement in a health intervention and what is the optimal number and combination of mechanics to be used in particular contexts. The optimal selection of mechanics likely depends on the goal of the task, its theoretical grounding, the researchers' and developers' approach, including what the players are meant to be doing, and for how long. Through our review, we aim to shed light on the interplay of some of these factors.

### 1.4 Is Applied Game Design effective?

Over the years and across reviews, the effects of Applied Game Design on outcomes have been mixed. Although it is clear that motivation and engagement are positively impacted by game-based interventions, their effects on targeted health outcomes vary (4, 23). Some studies find positive effects on motivation, with game-based tasks being more motivating to participants compared to non-game-based tasks (23). Uses of Applied Game Design seem to have an overall positive impact on health and well-being outcomes; for example, by helping individuals increase their physical activity–with 59% of studies reporting positive effects (4). However, there is still a large percentage of studies displaying mixed or null findings (4). As others have noted, null findings may have to do with limitations of the studies that can impact the efficacy of the intervention, which we address next (4, 16, 23, 24).

#### 1.4.1 Recent criticisms

One criticism concerns the varied methods used in designing and implementing game-based interventions. Some game-based interventions are delivered in more controlled settings, such as in the lab on a computer, while others are delivered on the participant's personal device; the number of training sessions also varies, with session durations ranging from a short five minutes to over an hour (23).

The quality of Applied Game Design studies also fluctuates greatly, ranging from being rated as having weak to strong methods (16). Many studies have small sample sizes (*N* < 50) and are too underpowered to evaluate the impact of the intervention. Studies also tend to report a lack of appropriate controls: many studies do not include a direct comparison between Applied Game Design methods and other types of intervention (4). This variability in method use and quality could be due to a lack of set procedural guidelines during the implementation and development of the studies (16).

The lack of interdisciplinary teams working on this research could also contribute to methodological weaknesses (16), as for example when researchers without design or technical background do not effectively integrate game elements in the intervention. Indeed, the wrong choice or combination of elements can tax cognitive load and frustrate participants (16). Using Applied Game Design effectively seems to require, not only more standardized procedural guidelines for its development and implementation, but also knowledge from both psychological and health science and game design (7, 16).

Past work has varied in its use of theories to inform design (16, 24). Researchers in Applied Game Design often draw from a wide array of psychology and health behavior change theories (24) to explain behavior and game design choices. However, the extent to which Applied Game Design studies ground their design choices in theory is often unclear. One issue is that, until recently, there have not been widespread frameworks for Applied Game Design to guide researchers in designing and implementing these interventions (16).

Psychological theory and game design theory relevant for Applied Game Design include frameworks around engagement, motivation, and behavioral drivers. Among psychology and health behavior change theories, developers of game-based intervention have used social cognitive theory (SCT), the health belief model (HBM) (20), social-determination theory (SDT) (5, 16) and behavioral economics (22, 25). Among game design theories, developers of game-based interventions have drawn from flow theory (26), goal setting theory (15), narrative transportation theory (27), the proteus effect (28), and AGILE development (29). These theories and concepts provide different perspectives on motivation, with implications about how to choose game and intervention components. For example, flow theory describes an intrinsic motivation coming from a state of intense focus driven by the enjoyment and challenge of a task (26). Behavioral economics posits human behavior to be driven by emotion and motivated by concepts of “loss” and “gain” (22, 25). AGILE development encourages the use of intended users in design choices and an iterative process (29). When applied to game design, flow theory is used to inform how to balance the challenge of the game with player abilities (21). Behavioral economics is often used to support the use of a reward and punishment system within the gameplay (22, 25) And the AGILE suggests that the best games are made by integrating knowledge of experts and players (29).

#### 1.4.2 Recent recommendations

Despite some mixed results regarding the efficacy of game-based interventions in health, most researchers still see the promise of game-based health interventions and have offered recommendations for how to improve its use moving forward (16, 23). The recommendations include using larger sample sizes, including control groups, using interdisciplinary teams, and better grounding the development of the game-based intervention in theory. There has also been a push for using more iterative, or cyclical, design processes during development that entails prototyping, testing, evaluating, and redesigning/refining (16, 23). Additionally, it has been suggested that development processes should be universalized (namely, through the use of a pre-development, development, and post-development phases) and that these development processes should use iterative design (16). These recommendations underscore the need to establish set guidelines or common practices for developing game-based health interventions.

### 1.5 The current study

This scoping review is focused on recent studies that use game-based interventions to improve health outcomes in order to evaluate common threads and gaps in this domain, specifically the current methods for developing game-based health interventions, patterns in design, and provide recommendations for future work. The goal is to aid in the future development of health technologies by providing insight into current design practices and recommendations. As the creation of game-based health interventions grows, there is a need for reviews in this work to reflect on methodology and guide improvements.

The purpose of this review is therefore three-fold. The first aim is to characterize common patterns in the development, assessment, and reporting of game-based health interventions. Common patterns in the mechanics used, prominent game design and psychological theories, and the methods used to support this design and implementation of game-based health interventions are considered. By evaluating the methods of applying game elements and mechanics used across studies, the hope is to identify common techniques that have been shown to be effective. By evaluating the theoretical grounding of research in this domain (namely, whether psychological theories of behavior change and theories of Applied Game Design are being applied), we can gain insights about decisions made during the design and the implementation of the intervention, since these can be motivated and informed by theory.

The second aim is to establish whether there has been improvement in this research area in recent years, given prior critiques, with researchers theoretically grounding their design choices, using robust empirical methods, and describing consistently and transparently their game development process.

The third aim, based on our findings, is to synthesize the current state of this literature and identify areas of growth to provide recommendations for researchers for improving the development, implementation, and transparent reporting of Applied Game Design work.

## 2 Methods

### 2.1 Article selection

A scoping review is an approach to reviewing the literature and synthesizing evidence used to examine concepts and methodology for complex areas of work. It entails assessing the current state of research, including the examination of gaps (30–32). The literature on Applied Game Design for health lends itself for a scoping review: the area is increasingly complex, studied across disciplines and health contexts, through different methodologies, which has been a criticism of the work (16, 24).

For this scoping review, the recommended procedures (30–32) and reporting guidelines of PRISMA-ScR (31) were followed. Both authors used their knowledge on the topic and reviewed the literature to devise the research question and develop the search criteria. Our criteria to identify relevant studies included: articles were written in English, published in the last 10 years, in which authors reported the development of their own intervention task, and targeted the improvement of a health outcome. Reviews and meta-analyses were excluded.

**Figure 1:**
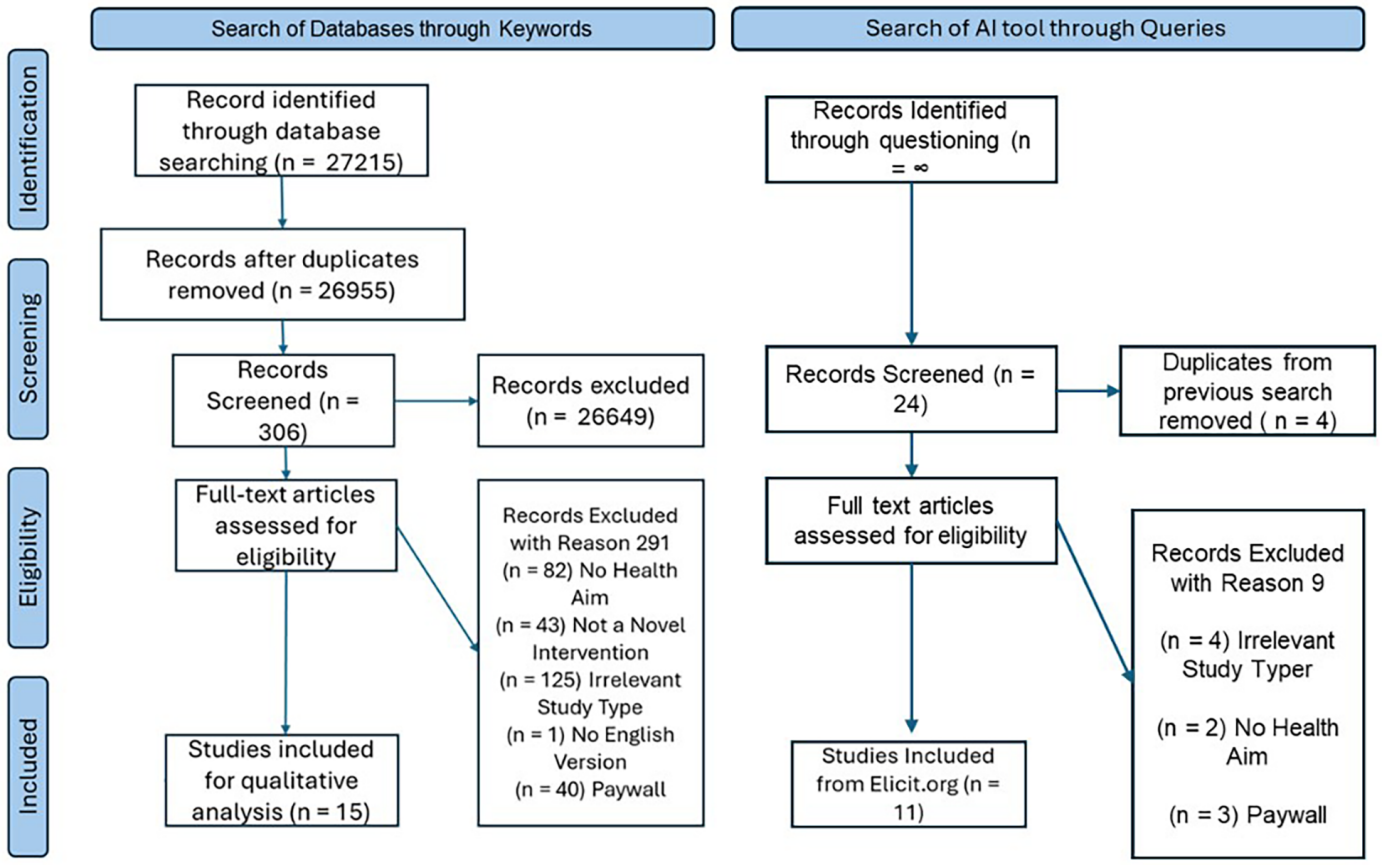
Scoping review process. These #s are estimation as performed by 2024 data search, and are approximations to mirror the 2023 search.

To select potentially relevant papers for the review, the following databases were used during February and March of 2023: ProQuest (PsycINFO and ERIC), Web of Science (Web of Science Core Collection), and PubMed, along with the online search engine Google Scholar. As a first step, the first author manually searched these databases using the following keywords: “health”, “health behavior change”, “gamification”, “serious games”, “health intervention”, “game design”, “game elements”, “gamification and health”, “simulation and health behavior change” and “health and gamification intervention NOT review”. Although we did not retain information about how many articles the initial search yielded, to estimate that number we conducted a search in October 2024 to demonstrate our process (see Figure 1). This yielded an estimate of *n* = 27,215 of articles initially obtained. After removing duplicates and filtering out any articles without key words such as “game”, “gamification”, and “virtual” in the title, we identified 306 relevant to our review. Then, by our inclusion criteria, 15 articles were identified to be accessible through our institutional licenses with publishers. Given our criterion that the researchers had to develop their own intervention and describe their development process, approximately 43 papers were excluded as the intervention was described in another paper.

As a second step, the tool Elicit was queried to expand our literature search to capture papers that were not identified by searching the traditional databases in the previous step. Elicit is a tool that uses large language models, and sources articles from multiple databases, to answer user question prompts about empirical research through searching the literature and identifying relevant articles (the version we used in spring 2023 is located at old.elicit.org). It requires a different search strategy–of using questions instead of keywords. The question prompts we used included “How does gamification impact health care outcomes?”, “How does gamification impact health outcomes in the long-term?”, “How does gamification impact health outcomes in the short-term?”, “What is the evidence for the efficacy of gamification in health interventions?”, “How are gamification health interventions made?”. Search results were filtered to include articles that were “randomized control trial” and “longitudinal” studies, and to exclude articles of other study types classified as “review”, “systematic review”, and “meta-analysis”. The resulting articles were screened based on the inclusion/exclusion criteria previously discussed. Because the Elicit tool produces an infinite scroll of results, it was not possible to quantify and document the search output. Seeing that Elicit still includes articles that are reviews and meta-analysis (as lower ranked items, farther down the results page), even when these are listed as exclusion criteria, we stopped looking at articles once the search output showed mostly review articles. Through this step, an additional 11 articles were identified (see Figure 1).

Finally, upon reading these selected articles, both authors confirmed that they met our criteria: that they were accessible with our institutional library's license, written in English, stated use of a novel intervention using applied game design, and reported having a health aim. Of the included articles, 21 explicitly reported designing their own intervention, while 5 articles seemed to be using a very similar intervention to one another article. Those 5 articles were included to be more inclusive in our selection, despite uncertainty about their distinctiveness, since each paper introduced a new condition or variant of the intervention.

This scoping review was limited in nature, since our investigation of the literature was not exhaustive. It is possible that, in addition to those articles behind a paywall that we excluded, relevant articles were missed due to our specific keywords, question prompts when searching the databases, and the architecture of the AI tool described above. More articles could be considered in a review that uses less strict inclusion criteria; in particular, one that does not require that the researchers developed their own intervention. In total, *n* = 26 articles were included for review.

### 2.2 Coding

For each of the 26 articles, data were charted (30–32) by coding along two main dimensions: (1) Development (theories invoked by the researchers to contextualize or motivate decisions surrounding the intervention, and the game mechanics that the intervention employed), and (2) Evaluation (the methods the researchers used to evaluate the intervention). To develop our coding scheme, qualitative methods similar to thematic and content analysis (30–32) were used, consistent with recommendations for scoping reviews. This approach is described below.

### 2.3 Coding of intervention development

#### 2.3.1 Theories

We coded for the explicit mention of (a) game design theories (e.g., flow theory, Proteus effect, etc.), and (b) psychological and behavior change theories (e.g., goal setting theory, social cognitive theory, etc.). To be coded as present, these theories needed to be described by the authors of the articles as supporting the development of the interventions. For each article, we re-read the background and methods sections and looked for explicit mentions of game design and psychological theories being used in the intervention development.

During coding, some points warranted discussion to refine our decision criteria. For example, from the outset it may be ambiguous whether goal setting theory constitutes a game design or a psychological theory. So, for goal setting theory, our determination was based on whether the authors described the theory as guiding a choice in the design of the game (game design theory) or as support for how aspects of their game impact human thought and behavior (psychological theory). We also made the decision to not code mentions of adopting a “gamification approach” as constituting a theory *per se*, since gamification is not a theory on its own. Although some researchers invoked the “gamification approach” and related constructs (e.g., “serious games”) in the motivation for their work, it was implicit that all researchers in our sampled articles used an applied game design approach. Therefore, references to a gamification approach were coded as a separate category, distinct from theories.

The first author coded all the articles, and the second author a subset of them (five articles or 19% of the sample). For the initial coding, the coders had 80% agreement on game design theories and 70% agreement on psychological theories. After discussion, coders came to 100% agreement for both theory types.

#### 2.3.2 Game mechanics

Game mechanics were classified as belonging to five main categories: reinforcement, immersion, performance, social components, and ecological components. Reinforcement encompasses rewards and punishments, including points, badges, medals, and feedback. Immersion includes the use of story or narratives, use of an environment for gameplay, and inclusion of an explorable area. Performance pertains to the use of goal setting, challenges, levels, and progress tracking. Social components include the use of competition, leaderboards, social pressure, collaboration, or communication with other players or an AI system. Ecological components include timed events, time limits, randomness or surprises, and use of marketplaces or economies.

We chose to use a different categorization than that of other researchers [e.g., (1)], reviewed in our introduction. Our categorization was derived in both a bottom-up manner, by surveying and grouping the game mechanics used by the authors, and a top-down manner, by consulting prior classifications [e.g., (1, 10, 11)]. All the unique mechanics terms noted by the authors were collected, then synonymous or functionally similar terms (e.g., badges, medals) were grouped together, forming semantically related categories. Lastly, following principles from thematic analysis and its iterative process, we distilled the category name for these groups of terms while consulting relevant articles and we refined these categories, as needed (30, 33). In some cases, we needed to clarify how we would apply these categories during coding, based on how these terms were used in the articles. For example, there was originally a separate category for Goals (which included goal setting, assigned challenges, levels, self-progression) and Progress (which included tracking) but these categories were combined into Performance (goal setting, assigned challenges, levels, tracking) because all these mechanics served similar purposes associated with advancement. Τhis choice was validated by the fact that Performance is a standalone category in some taxonomies [e.g., (11)].

In applying the coding scheme, we dealt with some additional ambiguities in classifying some mechanics (e.g., whether delayed rewards should be considered as falling under Reinforcement or the Ecological dimension), or deciding whether an aspect of the intervention should be classified as a mechanic (e.g., whether the dosing of intervention/gameplay is a game mechanic or a general intervention method). Upon discussion of these points, we made executive decisions and finalized our coding (e.g., to consider delayed rewards as Reinforcement and to not consider dosing a mechanic).

The first author coded all the articles, and the second author a subset of them (five articles or 19% of the sample). For the initial coding, the coders had 84% agreement in identifying game mechanics from the five key categories. Upon discussing and resolving the ambiguous cases above, agreement was 96% on identifying the same mechanics in these articles.

#### 2.3.3 Developmental approaches

Developmental approaches are those concerning the development of the intervention, the steps and methodologies used by researchers to create the game-based intervention. For developmental approaches, we coded for whether researchers used user-centered approaches (e.g., eliciting feedback/participant input, reporting engaging in a co-creation process with users, reporting being patient-centered), testing or pilot testing strategies (piloting an intervention, engaging in feasibility or acceptability testing), and an iterative design approach to integrate feedback (using iterative processes or multistage designs and methods to integrate feedback in the development of the intervention). These categories were derived using an inductive approach. Methodological terms used by researchers were collected to describe their study design and process used to inform the design of their interventions. Similar terms were then grouped together based on the definitions used by the researchers. Once grouped together, both authors decided on a category name to define each grouping.

We also coded the stage of development of the intervention, adopting the distinction made by Khaleghi and colleagues (16) to situate the intervention in the pre-development, development, or post-development stages. Studies were classified as predevelopment if the researchers conducted prototyping and feasibility testing while still in the planning phase of their interventions, or else stated their goal was to improve the initial development of the intervention. Studies were classified as being in the development stage if they were testing the efficacy of their intervention or testing the most effective implementation of their intervention. Studies were classified as being in the post-development stage if the researchers stated their intervention had already been determined to be effective, stated their goal was to test its efficacy in other populations, or if the study involved disseminating/applying the intervention. This coding was done by the first author based on the description of the study, its goals, and the description of its implementation.

Finally, we coded for a set of additional methodological features: the use of interdisciplinary research teams and the intervention duration (the length of intervention and the frequency of use of the applied game design tool).

As with the previous coding, the first author coded all the articles, and the second author a subset of them (19% of the sample). For the initial coding, the coders had 84% agreement and after discussion 100% agreement on the methods (research design and developmental) used by each study.

### 2.4 Coding of evaluations

For each study, we identified the methods used to evaluate the efficacy of the game based interventions. For evaluative methods we used a deductive approach and coded for commonly used research designs: namely, quantitative, qualitative, and mixed designs. The quantitative methods code encompassed studies that explicitly mentioned methods such as surveys/questionnaires, experiments, randomized controlled trials, cluster randomized trials, and the use of biological measures such as step count and blood pressure. The qualitative methods code encompassed studies that explicitly mentioned using interviews, focus groups, qualitative surveys, observation of video recordings, and thematic analysis. The mixed methods code applied to studies that described using both quantitative and qualitative methods, as defined above. Other evaluative tools used by researchers, such as observation of health outcomes, were not coded for and were instead recorded verbatim.

### 2.5 Data synthesis

After the coding for all articles was completed, and reliability determined, the codes were used to organize articles and draw patterns from the data. All codes were placed within a single spreadsheet along with descriptions and findings from the studies, in an expanded version of Table 1 [see the file in our OSF repository (34)]. Following the study aims, the first author organized the articles, grouping them together, based on their similarities between theory use, mechanic use, stage of development, and evaluative approach/design. This entailed creating a succinct table, pulling out information from articles with those similarities and having them in a format for easier comparisons. This permitted us to compare studies based within and across categories to extract potential patterns, including numerical ones.

**Table 1:** Summary of articles.

Paper title	Intervention	Population sampled & sample size	Study procedure	Findings
Mila Blooms: A Mobile Phone Application and Behavioral Intervention for Promoting Physical Activity and a Healthy Diet Among Adolescent Survivors of Childhood Cancer^a^	This intervention was designed to improve weight management. Participants play as an avatar in a team to complete eight expeditions. To progress through the game each participant has to earn points through physical activity and diet tracking. Each expedition also has a set of challenges to learn and practice diet management skills.	Parent/child (12–17 years of age) dyads from two pediatric oncology clinics, *N* = 15	Quasi experimental single-group pretest/posttest design	Participant satisfaction feedback indicated ease of use and enjoyment of the app. Despite limited evidence for behavior change, this was taken as a demonstration of the viability and appeal of the game features with no adverse side effects. There was an observable significant decrease from pretest to posttest in physical activity, an increase in percent time in sedentary activity, and an increase in healthy eating self-efficacy. For behavior change, there was a decrease in sugary beverages and maintained sedentary activities. There were difficulties addressing technical bugs, additional costs, and time required to develop the backend dashboard.
Developing a Serious Videogame for Preteens to Motivate HPV Vaccination Decision Making: Land of Secret Gardens^a^	This intervention was developed to provide education on HPV. The goal is to plant a lush secret garden and protect the seedlings by treating them when they sprout with a potion to keep them healthy as they mature. Points to buy seeds and create the potion are earned by playing mini-games. Throughout the process of play, players are exposed to messaging about HPV and the benefits of the vaccine.	Preteens (11–12 years of age), *N* = 9	Focus groups	Participants were very willing to discuss their knowledge regarding HPV and vaccination. Knowledge gained was used to finish app development. The app was determined to be feasible.
Wellness Tribe: Gamification of the IS-WEL Adlerian-Based Model of Wellness^a^	This intervention targeted improving overall wellness. Participants use their group (or “tribe”) to offer and elicit support, feedback, and problem-solving in their wellness efforts. They self-assess their current levels of satisfaction in each element of the Wellness Tribe (the Sage, the Scholar, the Warrior, and the Alchemist) and determine behaviors that they would practice. Behavioral goals and a point system are developed by the group. Participants track their points and decide their rewards for leveling up. Groups can compete for points across elements.	counselors in training at an accredited university program in the southeastern United States, *N* = 30	Qualitative Case study	There was increased cohesion and cooperation in the classrooms. Students reported decreased mood-treating medication prescriptions, interacting actively with course materials, and an increase and attention to self-care, nutrition, and life balance.
Can a serious game-based cognitive training attenuate cognitive decline related to Alzheimer's disease? Protocol for a randomized controlled trial^a^	This intervention aimed to improve various cognitive functions. Participants play as a sailor visiting multiple islands. Each island represents a mini game to train one of three key cognitive domains specifically affected by AD (episodic memory, semantic memory and spatial abilities) and WM.	elderly participants from cognitively normal individuals at risk for AD with SCD to MCI mild AD patients, *N* = 162	bi-centric, randomized, placebo-controlled, within and partially blinded three arm design	No results yet
Machine Learning and Serious Game for the Early Diagnosis of Alzheimer's Disease^a^	This intervention aimed to improve cognitive decline in those with dementia. Online virtual simulation of common daily tasks to test working memory, episodic memory, executive functions, visuo-spatial orientation, concentration, attention, and language. Tasks included a navigation task (follow arrows to supermarket location), shopping task (list of needed items, minigames to earn money), kitchen task (organization of cooking steps), and a gardening task (select flowers from garden to water).	Elderly between ages of 60 and 84, *N* = 36	supervised Machine Learning algorithms to evaluate the ability of AlzCoGame to correctly predict healthy subjects and subjects with cognitive impairment or early Alzheimer's disease	The results and tasks of the game were deemed generally satisfactory and acceptable. Most participants found following the arrows in the navigation task difficult to get to the supermarket and found the memorization requirements of some tasks to be too difficult. The game resulted in 92% accuracy for diagnosing dimentia.
Unlock Me: A Real-World Driven Smartphone Game to Stimulate COVID-19 Awareness^a^	This intervention was developed to provide education on COVID-19. Players play as an enforcer and aim to catch violators of COVID-19 protocols within the given time frame by creating checkpoints in the game map. While patrolling, the player might encounter COVID-19 bombs. The game has a feature of collecting sanitizers that players can use to regain health points and receive a temporary shield that protects the player from violators and COVID-19 bombs. Throughout the game, information about COVID-19 is provided.	Professors, researchers, university students, *N* = 207	Iterative 4 phase design (discover, define, develop, deliver). A post-game questionnaire and evaluation model were used to assess prototyping.	An observed increase in learning by 53%. About half of the players liked the Aesthetics; Just over half felt that the game was easy to learn; just over half agreed that the game was operable; a little under half agreed that the game was accessible; just over half felt confident and had a feeling of mastery after playing the game; just over half felt satisfied with the game and had a feeling of accomplishment; a little under half found the game to be fun to play; a little under half were immersed; just over half found the game to be relevant; over half felt they had learned something.
Assistive HCI-Serious Games Co-design Insights: The Case Study of i-PROGNOSIS Personalized Game Suite for Parkinson's Disease^a^	This intervention was developed to improve physical activity. i-PROGNOSIS uses a gamified environment based on a personalized approach that involves different serious games, including ExerGames, DietaryGames, EmoGames, and Handwriting/Voice Games, all integrated under a unified platform.	Parkinson's Disease patients, health care professionals, researchers, *N* = 104	Web survey, semi-structured interviews	Various game mechanic parameters (e.g., clear rules, being able to complete goals, enough options, interesting options, enough surprises) were predictive of participants’ evaluation of how important they were for transferring the skills targeted by the games to real life. The factors that were significant differed somewhat by type of game. The semi-structured interviews also gave some insights about how game design features could be refined.
Using Serious Games for Antismoking Health Campaigns: Experimental Study^a^	This intervention was designed to discourage smoking. A flash-based single-player game where users are asked to avoid smoking cigarettes when they are stressed out because of an upcoming exam (Level 1) and when they are hanging out at a bar (Level 2).	Undergraduate students who reported smoking within the last 30 days, *N* = 72	Experimental 2 × 2 between-subjects design	Individuals in the game condition reported significantly more negative attitudes toward social smoking. There was no significant difference in the intention to quit smoking between the game condition and the print condition. Individuals in the game condition reported a greater level of susceptibility than those in the print condition. The study found that when smokers play the game, they experience more negative attitudes toward social smoking and greater susceptibility than those who read the pamphlet.
Improving instruction and sexual health literacy with serious games and gamification interventions: an outlook to students’ learning outcomes and gender differences^a^	This intervention was designed to provide education on sexual health. The scenario game comprises five topics on sexual health promotion for adolescent students. The game scenarios were presented as avatars of students and a teacher having a conversation about the taught topic. They then completed quizzes.	regular students from a lower secondary school, *N* = 108	quasi-experimental research design	For all three interventions (normal, use of reward and competition, simulation gameplay) resulted in improvement from pre to post-test. Students in the simulation game and reward/competition arms showed more improvement (no significant difference between the two).
Treating Childhood Social Anxiety Disorder With Virtual Environments and Serious Games: A Randomized Trial^a^	This intervention was developed to reduce social anxiety disorder. A virtual environment with an entire school and outside areas with free roam. There were 8 dynamic avatar characters that participants could engage with to receive therapeutic guidance.	Children between 7 and 12 years with a diagnosis of SAD, *N* = 43	non-inferiority design, feasibility study; experiment	Both programs were equally efficacious in decreasing anxiety and improving social skills in social encounters. Children and clinicians were satisfied with the technology and rated it as credible and easy to use. Parents were satisfied and indicated that they would recommend it to their family and friends. Those who used the game showed as much improvement as children who received the gold-standard treatment. Both groups made statistically significant improvements from pre- to post- treatment based on self-report, parental report, clinician ratings, and behavioral assessments. Parent-reported child social anxiety was better for the game group. 60% of children treated with Pegasys-VR™ no longer met diagnostic criteria for SAD.
The Feasibility and Acceptability of Virtual Environments in the Treatment of Childhood Social Anxiety Disorder^a^	This intervention was created to help reduce social anxiety disorder. In a school setting; practice four areas of social skill: greetings and initiating conversations, maintaining conversations through asking questions, giving and receiving compliments, and assertiveness. 3 levels; 744 unique dialogue responses	Children between the ages of 8 and 12 diagnosed with SAD, *N* = 11	Feasibility study	Feasibility: 90% had access, 36% needed technical support, 27% needed to borrow technology. Acceptability: 73% completed, 75% would recommend, 3% skipped some at-home sessions. Credibility: 100% rated it as logical in decreasing anxiety, 88% believed that the treatment would specifically help them become less anxious, 75% also reported this treatment as helping them improve other areas of their functioning.
Stigma-Stop: A Serious Game against the Stigma toward Mental Health in Educational Settings^a^	This intervention was designed to provide education to reduce mental health stigma. The video game presents four of the most common disorders among young people. Players can interact with 4 characters each with one of the 4 disorders. The objective of the player is to convince the characters to work toward a common goal, which is to participate in a video game design contest. This brings the player to minigames that reference a mental illness and allows the user to learn information about each particular disorder	University Students, *N* = 552	Experiment; pre/post test	Stigma was shown to decrease in those who played the game. Self-efficacy to help others also demonstrated improvement. Participants scored close to eight points (7.8) for the game's usefulness and a slightly lower score for interest (average score of 6.3); 75% stated they would recommend trying this game to a friend
Methodology of an exercise intervention program using social incentives and gamification for obese children^b^	This intervention was designed to improve physical activity. WeChat was created for each group. There are weekly activities including peer support, daily reports, a weekend competition, and tip reading.	Obese Children, *N* = 420	Experiment; intervention and control groups	No results yet
Mobile health-based gamification intervention to increase physical activity participation among patients with coronary heart disease: study protocol of a randomised controlled trial^b^	This intervention was designed to improve physical activity. Gamification WeChat applet named “TahneeWeh” with weekly challenges, step tracking, and educational content.	18–70 years and diagnosed with CHD, *N* = 108	Single-blinded three-arm randomized controlled trial	No results yet
The effect of an online exercise programme on bone health in pediatric cancer survivors (iBoneFIT): study protocol of a multi-centre randomized controlled trial^b^	This intervention was developed to improve bone health and physical activity. Whatsapp game with instructional content and tracking and feedback to encourage exercise, specifically jumping.	Pediatric cancer survivors aged 6 to 18 years, *N* = 116	Multi-centre, parallel groups RCT	No results yet
The effect of social support features and gamification on a Web-based intervention for rheumatoid arthritis patients: randomized controlled trial^c^	This intervention was designed to improve physical activity. A website with rewards given for interaction with website components such as educational content and a chat.	Patients with a diagnosis of RA, *N* = 157	Randomized control trial	Compared to the control group, physical activity over time increased and health care utilization decreased for patients with access to social support and the game. Gamification increased the use of the website.
Gamification as an approach to improve resilience and reduce attrition in mobile mental health interventions: A randomized controlled trial^c^	This intervention was designed to improve overall wellness. eQuoo is a cognitive behavioral therapy journal app providing psychoeducational tutorials, choose-your-own-adventure scenarios to teach and allow players to practice applying skills.	Adult employees in a wellness program; *N* = 358	Randomized control trial	Compared to the 2 control groups, significant increases were seen in resilience, personal growth, positive relations, and reduced anxiety in the gamification app group. The app group also had more adherence than the control or waitlist groups.
Smartphone-based gamification intervention to increase physical activity participation among patients with coronary heart disease: intermediate results of a randomized controlled trial.^c^	This intervention was designed to improve coronary heart disease through increasing physical activity. An applet called “TahneeWeh” that used points and levels to reward step counts	Patients between the ages of 18 and 70 years, diagnosed with CHD and received PCI treatment during hospitalization; *N* = 108	A single-blind, randomized, controlled trial with three arms	For the individual group, the intervention significantly increased PA among CHD patients and had a good maintenance effect during follow-up. Patients also had a significant increase in competence and autonomous motivation and a significant decrease in BMI and waist circumference. For the team group, gamification intervention with collaboration didn't result in significant increases in PA. Patients in this group had a significant increase in competence, relatedness, and autonomous motivation. The game design did not increase patients’ autonomy.
Effect of Goal-Setting Approaches Within a Gamification Intervention to Increase Physical Activity Among Economically Disadvantaged Adults at Elevated Risk for Major Adverse Cardiovascular Events: The ENGAGE Randomized Clinical Trial.^c^	This intervention was designed to reduce the risk of cardiovascular disease through increasing physical activity. Way to Health website, weekly step goals, and daily feedback.	Participants in lower-income neighborhoods; *N* = 500	Randomized clinical trial	The control arm had a mostly steady increase in physical activity from baseline. Three of the gamification arms also had mostly steady increases in physical activity. The gamification arm with self-chosen and immediate goals had the largest increase in physical activity.
Effect of a Game-Based Intervention Designed to Enhance Social Incentives to Increase Physical Activity Among Families: The BE FIT Randomized Clinical Trial^c^	This intervention was designed to increase physical activity. Using Way to Health platform, participated with family, weekly challenges, lifelines, and levels.	Adults already enrolled in the Framingham Heart Study; *N* = 200	Randomized clinical trial	The percentage increase in participant step goals from baseline was not significantly different between study arms. The proportion of achieving step goals remained constant throughout the intervention period for both study arms but declined for the gamification arm during the follow-up period. Those in the gamification arm achieved step goals on a significantly greater proportion of participant days. The gamification arm also had a significantly greater change in the mean daily steps than the control arm. Most participants in both study arms had positive perceptions about their experiences in the study. Physical activity levels among participants in the gamification arm declined over time but overall remained significantly greater than those in the control arm.
Effectiveness of Behaviorally Designed Gamification Interventions With Social Incentives for Increasing Physical Activity Among Overweight and Obese Adults Across the United States: The STEP UP Randomized Clinical Trial.^c^	This intervention was developed to help with weight management. Used Way to Health website, daily step goals, levels, and collaboration/competition	Deloitte Consulting employees with a high BMI; *N* = 602	Randomized clinical trial	The gamification with competition arm had the highest physical activity levels during the entire trial. The other gamification arms had more average daily steps compared to the control. Compared with controls, participants had a significantly greater increase in mean daily steps from baseline. Gamification with competition resulted in the greatest increases in physical activity and was the only intervention for which physical activity remained significantly greater than that for the control group during the follow-up period.
Effectiveness of a gamification strategy to prevent childhood obesity in schools: a cluster-randomized controlled trial^c^	This intervention was used to help with weight management. The game involves challenges (target eating habits, steps, and other activities), points, completed with family, a leaderboard, real-world rewards, and an online platform to monitor progress.	The student population were children in fifth and sixth grade in schools in the neighboring municipalities of Santiago and Estación Central in Santiago, Chile; *N* = 2,197	Longitudinal cluster controlled trial	The intervention arm had a lower BMI score than the control arm after adjusting for school and individual covariates and baseline values. The waist circumference was similar between the intervention and control arms. Students in the intervention arms experienced a reduction in their BMI compared with controls and systolic blood pressure, diastolic was similar between intervention and control arms at 7 months.
Effectiveness of a Text-Based Gamification Intervention to Improve Physical Activity Among Postpartum Individuals With Hypertensive Disorders of Pregnancy: A Randomized Clinical Trial.^c^	This intervention was designed to help reduce the impact of hypertension through increasing physical activity. Team-based gamification intervention consisting of points and levels and leveraging the performance of other participants on the team. All participants received a wearable activity tracker, established a baseline step count, and selected a step goal greater than the baseline.	Postpartum individuals with a recent pregnancy complicated by a HDP; *N* = 127	Randomized clinical trial	Participants in the intervention arm consistently had a higher step count compared with those in the control arm. Those in the intervention arm achieved their step goals on 47% of days compared with 38% for the control group. Among participants who completed the end-of-study survey (81.1%), most would recommend the study to others (94.2%)
Remotely Monitored Gamification and Social Incentives to Improve Glycemic Control Among Adults With Uncontrolled Type 2 Diabetes (iDiabetes): Protocol for a Randomized Controlled Trial^c^	This intervention was designed to help individuals with diabetes with their weight management. Participants randomized to 1 of the 3 gamification arms conducted goal setting, weekly weight target, daily step goal, and an HbA1c goal. Points and levels are used with social, competitive, and collaborative components.	Aged between 18 and 70 years who had a diagnosis of type 2 diabetes with a high HbA1c level; *N* = 361	A 4-arm randomized controlled trial with a 1-year intervention period	This trial has demonstrated that it is feasible to conduct a remotely monitored intervention – no data analysis yet
Efficacy of gamification-based smartphone application for weight loss in overweight and obese adolescents: study protocol for a phase II randomized controlled trial^c^	This intervention was developed to improve weight management. The application comprises tracking and monitoring functions using diaries of food intake, exercises, and daily steps. It uses challenges and points systems to promote healthy nutrition choices and exercises. Weekly reminder texts and monthly motivation interviews are also given.	Overweight obese adolescents in primary care; *N* = 108	Phase II, single-center, two-arm, triple-blinded, RCT	No results yet
Evaluating the Impact of Adaptive Personalized Goal Setting on Engagement Levels of Government Staff With a Gamified mHealth Tool: Results From a 2-Month Randomized Controlled Trial^c^	This intervention was developed to increase physical activity. Using GameBus, a health promotion campaign was specially designed to promote walks, bike rides, and sports sessions. Challenges, points, goals, progress visualizations, leaderboards, and rewards were used.	Staff members of 7 governmental organizations in the region of Antwerp, Belgium; *N* = 176	2-arm randomized intervention trial	Engagement was higher for participants who had set themselves a goal in the intake survey. Personalization seems particularly promising for promoting the frequency of physical activity. The treatment group participants who set themselves a goal reported the lowest levels of self-efficacy on average among all participants.

See a full table of our data within OSF and the Supplementary Materials.

^a^ Represents the paper was found using PsycINFO.

^b^ Represents the paper was found using PubMed.

^c^ Represents the paper was found using Elicit.

### 2.6 Data availability

Our OSF repository (34) for the project makes available the full coding of these articles, including whether the researchers developed their own intervention, their targeted health outcome, the search engine the article was located with, and our coding as described above. Our coding includes additional information for each article, including the goal of the intervention, the duration of the intervention, a summary of the intervention task, and key results. This study was not preregistered.

## 3 Results

Our evaluation approach involves summarizing frequency distributions of the dimensions coded to gauge the prominence of game mechanics, theories, and methods used, followed by qualitative observations to contextualize these results.

### 3.1 Development

#### 3.1.1 Prominence of theories

As shown in Table 2, most articles referred to psychological theories: in total, 21 articles (80.8%) described their interventions as being supported and guided by one or more psychological theories. Out of these, 10 (38.5%) also referenced game development theories. On the other hand, less than half of the articles (42.3%, 11 articles total) referred to game development theories, suggesting that the grounding of these interventions in game development theory is lacking. This is despite the fact that the overwhelming majority of the articles did superficially reference terms such as “gamification” or “serious games” (92.3%, *N* = 24) to support their use of a game-based intervention. Strikingly, a subset of articles (15.4%) did not reference any specific theory to support the development of their interventions.

**Table 2:** Summary of results.

Dimension coded	Number of articles (frequency)
Theory	
Game development only	1 (3.8%)
Psychology theory only	11 (42.3%)
Both game development and psychology	10 (38.5%)
Neither	4 (15.4%)
Number of mechanic categories	
1 category	0 (0.0%)
2 categories	4 (15.5%)
3 categories	13 (50%)
4 categories	4 (15.5%)
5 categories	5 (19.2%)
Mechanic categories used	
Reinforcement	23 (80.5%)
Immersion	14 (53.8%)
Performance	23 (80.5%)
Social	20 (76.9%)
Ecological	8 (30.8%)
Game development methods	
User-centered	11 (42.3%)
Testing	14 (53.8%)
Feedback integration	8 (30.8%)
Stage of development	
Predevelopment	5 (19.2%)
Development	11 (42.3%)
Post development	10 (38.4%)
Methodological approaches	
Quantitative only	15 (57.7%)
Qualitative only	3 (11.5%)
Mixed methods	8 (30.8%)

The psychology theories being used in the development of game-based interventions focused primarily on motivation and drivers of behavior. Of the 21 articles referring to psychological theories, the most pervasive theory was behavioral economics (38.1%), followed by self-determination theory (28.6%), and health behavior change theories (19.1%). In the 11 articles that mentioned a game design theory, few game design theories were referenced consistently, with the exception of user-centered design approaches, which was referenced in almost half of the articles (*n* = 5, 45.5%).

#### 3.1.2 Prominence of mechanics

As shown in Table 2, all interventions used at least two categories out of the five major categories of mechanics (Reinforcement, Immersion, Performance, Social components, Ecological components), with half of the articles using three categories. About a fifth of the articles included all five mechanic categories.

As shown in Table 2, the most popular categories of mechanics were Reinforcement (points, badges, punishment, etc.) and Performance (goal setting, challenges, etc.), both of which relate to game progression and a sense of achievement through rewards and accomplishing goals. Each of these two types was leveraged by the majority of the interventions (*N* = 23, 88.5% of articles). The least used mechanic category involved Ecological components (*N* = 8, 30.8% of articles), such as timed events, marketplaces, and randomness. Social components (e.g., teams, leaderboards) were also commonly used (*N* = 20, 76.9%) and so was Immersion through story and explorable environments, to a lesser extent (53.9% of articles).

We also noted that reinforcement, performance, and social components were most commonly used together (in 68.4% of articles), in line with past findings that these mechanics are used most frequently (1). The co-occurence of these three mechanic categories could be due to their baseline prominence in games in general, as well as the ease of their implementation, their known impact on motivation, and their connection to psychological theory. The connection between the co-occurence of these three mechanic categories and theory is discussed in the synthesis subsection.

#### 3.1.3 Prominence of design approaches

As Table 2 suggests, the most common method used for developing and improving game-based interventions across studies involved testing strategies (53.9% articles), which included piloting, feasibility, and acceptability determinations by the authors. Many articles (42.3%) also recruited user-centered design approaches such as co-creation which brings in potential users to ask their opinion and give feedback on development. The least common developmental method was feedback integration: in fewer than a third of the articles (30.8%) did the research team use multiple stages and iterative approaches to incorporate feedback into the development process. This may suggest that researchers don't seek out user feedback frequently enough or that the feedback in the design process is obtained but is underreported. Only 34.6% of articles (*N* = 9) explicitly mentioned using an interdisciplinary team to develop the game-based application.

As shown in Table 2, most articles reported game-based interventions that had passed the predevelopment stage. This suggests that most reviewed studies developed or implemented an intervention that had already undergone some initial pre-development by the research team. There were similar numbers of articles at the development and post-development stage.

### 3.2 Evaluation

As shown in Table 2, to evaluate game-based interventions, most articles used only quantitative methods (57.7%), followed by 30.8% of studies using a mixed methods approach including both quantitative and qualitative methods. A small portion of studies (11.5%) used exclusively qualitative methods.

The majority of studies (*N* = 20, 76.9%) included control or comparisons groups. Of these studies, 14 had results to report: 9 reported more positive outcomes for participants using the game-based interventions and 5 reported similar outcome effects of game-based interventions and non-gaming interventions. There are no observable differences (e.g., in theory or mechanics use) between those studies that found better rather than similar outcomes for game-based interventions vs. the comparison group. The rest of the studies that did not include a control group (*N* = 6) reported positive outcomes following game-based interventions for targeted health behaviors, such as increases in physical activity, improvement of mental health, and increased education.

Of those studies that reported results (*N* = 20), 10 also reported data on feasibility and acceptability of the intervention. Across these 10 studies, users overall reported enjoying the game-based interventions with most stating they would recommend use of these games to others. However, in some studies, users did report finding the game-based interventions difficult due to various aspects of gameplay and user-interface interactions. Multiple studies also reported technical issues, which negatively impact user experiences.

### 3.3 Synthesis: development

#### 3.3.1 Patterns around theoretical framing

One of the key aims of this review was to answer questions regarding the interplay between the theoretical grounding of the research studies and the game mechanics used. We found that articles that only referenced psychology theory were most likely to use the three mechanics of Reinforcement, Performance, and Social components together (91%, 10 out of 11). In contrast, articles that referenced game design theories (on their own and with psychological theories) were more likely to include the mechanic categories of Ecological components and Immersion (55%, 6 out of 11). When articles invoked both game design and psychology theory types (10, 38.5%) they were more likely to use a confluence of game mechanics (4 mechanic categories on average). Among these articles, Performance (e.g., the use of levels and challenges) was the most consistently used mechanic category, referenced in all of them. Among the remaining articles (which had one type of theory or no theory), Reinforcement was the most consistently used mechanic category (in 14 out of 16 articles; 87.5%).

A correspondence was also observed between theory use across stages of game development. In the predevelopment stage, no noticeable pattern of theory use emerged. However, in the development stage, we noted that 54.6% of studies referenced one type of theoretical backing, the majority being psychology theories (83.3%, in 9 out of 11 articles). In the postdevelopment stage, studies most commonly invoked psychology theories alone (50%) or in conjunction with game design theories (40%).

In terms of the use of interdisciplinary teams, we also noted that more than half of the explicit mentions of interdisciplinary teams (55.6%) were in articles that invoked both theory types. This is in line with the idea that interdisciplinary teams conducting research are more likely to incorporate theoretical perspectives from different fields.

#### 3.3.2 Patterns around developmental approaches

Another aim of the review was to explore the correspondence between the stage of the development of the intervention, the methodological approaches used (quantitative, qualitative, mixed,) and the developmental methods used to develop and evaluate the game-based intervention (user-centered, testing, feedback integration). There were consistent patterns between stages of development and methods used. All studies in the predevelopment stage combined quantitative methods with 1–4 developmental approaches. Every study in the predevelopment stage reported using testing methods or some form of prototyping.

Studies that explicitly mentioned using an interdisciplinary team all reported using testing methods and most (77.8%) reported using user-centered methods. The high use of testing and user-tested methods in these interdisciplinary collaborations is notable, since these development approaches are most common in design-oriented fields but are less common in psychology and health-oriented disciplines.

#### 3.3.3 Patterns around mechanics

In terms of mechanic choices, we observed that for studies in the development stage, Social and Reinforcement were the most popular mechanic categories, with 36.4% of studies using one or the other, and 63.6% of studies using them together. Studies in the post-development stage used three or more mechanics categories with every post-development study using both Reinforcement and Performance. The Social mechanic category was also commonly used within the post-development stage (80%, 8 out of 10). The prevalence of Reinforcement across stages suggests that it is a common and effective mechanic category. Studies within the predevelopment and postdevelopment phase tended to report using more mechanics than studies in the development stage (4 on average compared to 3 on average).

We also considered whether the involvement of researchers from multiple disciplines in the design process would lead to more varied use of mechanics in intervention development. Studies that explicitly mentioned using an interdisciplinary team incorporated three or more mechanic categories in their intervention. This could reflect the role of interdisciplinary teams on the design process, as well as the recruitment of more diverse theories (which in turn are associated with different types of mechanics, as we established).

### 3.4 Synthesis: evaluation

Patterns around evaluative methods were primarily found across development stages. Quantitative methods appeared to be mainstream during the development stage: all studies in the development stage (100%) included quantitative analysis, with 45.5% reportedly only using quantitative methods without describing other qualitative methods such as interviewing. The remaining 54.6% of studies in the development stage varied in their method usage, combining multiple quantitative and qualitative approaches (use of mixed methods).

Studies in the post-development stage used three or fewer methods, except for one study that used 4. The two most popular methods used in the post development stage were use of questionnaires or pre-test/post-test designs (quantitative, 80% of studies), and interviews or focus groups (50% of studies).

As expected, studies in the predevelopment stage more commonly used mixed methods (80%, 4 out of 5) and used more varied individual types of methods than those in development and post development, whereas studies in the post development using the least. Studies in the predevelopment stage also seemed to focus on feasibility and prototyping (leaning towards qualitative approaches), while studies in the post development stage focused on outcome measures and user feedback (quantitative).

## 4 Discussion

The aim of this scoping review was to examine the use of theory and methods in the development of game-based approaches to health interventions. Specifically, we examined how research in this domain is typically implemented, how research methods and game features vary according to the game design and psychological theories adopted by the authors, and the extent to which these recent studies addressed prior concerns and recommendations. Past reviews have criticized research using game-based interventions for their inadequate methods including the use of small sample sizes, the lack of comparison groups, and the disconnect from real-world testing (4, 19). Khaleghi and colleagues (14) issued recommendations for what to look for when evaluating research in this domain and how to properly utilize game-based approaches using sound methods and theoretical backing. In light of our results, we add to this set of recommendations for future research.

### 4.1 Key findings of the current review about the methods, mechanics, and theories used in health interventions

One of our key findings was that all studies we considered used more than one mechanic; most, in fact, used three types of mechanics. This is in line with other work that has reported that intervention studies include 2–9 mechanics (19). It suggests that the inclusion of only one mechanic may be insufficient to engage and motivate players intrinsically and extrinsically. Using a categorization system based on prior studies (1, 24, 26), we found that the use of Reinforcement and Performance were the most popular types of mechanics, consistent with findings from past reviews (3, 19). The prevalence of these mechanics may suggest that the use of rewards and progress-oriented aspects in interventions are believed to be effective motivators or are easier to implement.

Another key finding was that the theoretical grounding of intervention studies was associated with different patterns in their use of game mechanics. Specifically, those studies that referenced using both game design and psychology theories tended to include more types of mechanics. This suggests that incorporating theories from the field of game design may compel researchers to use other mechanics types, such as Immersion and Ecological components. Such diversification of mechanics makes sense given the game design theories researchers drew from to support their design choices (e.g., human-centered design principles).

In contrast, those studies that referenced only psychological theories tended to report the same three mechanics types (of Reinforcement, Performance, and Social components). This may suggest that an emphasis on psychological theories may compel researchers to leverage findings about positive reinforcement and social drivers as good motivators for behavior change, reflected in the mechanics chosen. Reinforcement and performance are mechanics that are directly related to the constructs of loss, gain, and optimization, directly related to the most common psychological theory referenced: behavioral economics (22, 25).

From our data, it was not possible to determine whether there is an optimal number or combination of mechanic categories for effective game-based interventions. There was no observable pattern between the number or the category of mechanics used and other variables we coded for. As we suggested earlier, the optimal number and category of mechanics are likely reliant on the targeted health behavior and the context in which individuals are using the intervention.

### 4.2 Revisiting criticisms

One of our aims was to evaluate whether recent works in the creation and testing of game-based health interventions have addressed prior criticisms. During our review, we noted reporting and transparency habits, empirical methods used, and theoretical grounding.

#### 4.2.1 Reporting

Reviewed articles were from an array of fields and empirical journals, including psychology, health, and game design (see Table 1). As such, we noted a wide array of reporting standards. While some studies detailed the development of their interventions and intervention components, others gave brief overviews of the interventions and did not address the process of development. For example, details regarding the interventions, such as the game mechanics used, were often underreported, which required us to infer that information from the intervention descriptions. In some cases it was also unclear whether the article was the initial publication introducing the developed intervention, or whether previous published articles had reported its development (e.g., such that pilot testing had been previously performed).

There were also differences among studies in reporting the motivation for the design of their intervention. With respect to providing theoretical background to motivate design choices, some studies did not report any theoretical backing at all, and most drew only from psychological theory, with no reference to game development theory or models. Studies also varied in the sense in which terms for different game components (e.g., “points” and “rewards”) were used. For example, some authors used “rewards” to refer to points in the game, whereas others used the term to refer to real-world rewards.

In additional coding reported in our supplement, we also determined that the majority of studies we reviewed did not explicitly state how long it took participants to complete the tasks or suggested that time using the tool was user-dependent and primarily self-guided. This paucity of information about the duration of the information makes it hard to determine the dosing that leads to the most efficacious results (19).

#### 4.2.2 Study designs

Overall, there was progress regarding prior methodological criticisms. One prior criticism was that there was a dearth of comparisons of game-based interventions to controls or other non-game-based interventions (23). As documented in the full coding reported in our supplement, the majority of the studies had comparisons to controls or other interventions. Most of these studies reported positive results of the intervention. Some of the studies obtained mixed results, in which game-based treatments and active controls had similar, positive effects on health outcomes and were noted to be acceptable by participants. In these studies, the authors reported that this may be due to challenges with development and implementation of the game-based intervention. Design is an iterative process, so such mixed results should lead to adjustments at the development stage of the intervention (14), which can result in future positive effects of the intervention.

#### 4.2.3 Generalizability

Another criticism of this literature has been the limited generalizability of game-based interventions, with many studies using in-lab testing or small sample sizes (19). Our review shows improvement on this front. Many of the studies we reviewed delivered their interventions outside of controlled settings as described in their methods (see our Supplementary Material): interventions were being tested or implemented in real-world settings such as at home, in classrooms, and in unconstrained settings through participants' mobile devices. The majority of these studies also delivered interventions with long-term potential, designed to be used repetitively over time. This permitted observing the impact and use of their game-based interventions over a protracted period of time (some spanning as long as a year). The average sample size across these studies was also higher than in previous reviews (4), averaging approximately 180 participants (after removing an outlier study that had reported over 2,000 participants). These larger sample sizes are important for adequately detecting the expected modest effect sizes of interventions in studies that included comparisons with control groups or alternative intervention methods (4, 16, 19, 23). Additionally, we noted that multiple studies in our review were in the post-development stage, implementing interventions with the people that need it or modifying interventions to fit target groups. This is yet another methodological improvement in that interventions tailored to targeted populations and delivered in real world settings are expected to have greater ecological validity.

### 4.3 Coding challenges and limitations

Throughout the review process we did come across multiple challenges in coding various aspects of these studies. This was in part due to variability in reporting standards and the amount of detail each study offered regarding the game-based intervention, consistent with earlier criticisms of this literature (4, 14, 19). This variability posed a challenge in coding for the mechanics, methods, theories used, and the intervention's development stage. As stated in Section 4.2.1, the reporting of these components was sometimes unclear or absent. Mechanics were not always explicitly mentioned, components of the methodology were often missing, and stages of development were not always described.

Our classification of the development stage of the game-based intervention does not mean the authors did not engage in predevelopment or postdevelopment, but that they may have published this information elsewhere without making that clear in the article at hand. Coding for the research methodology (quantitative, qualitative, or mixed) was relatively straightforward; however, there were still instances where due to underspecification in reporting, it was unclear whether particular sources of data (e.g., video recordings) were in fact used for qualitative or quantitative analyses.

Regarding the coding the theoretical frameworks used, as previously noted, some articles did not discuss the theoretical backing of the game-based intervention. Almost all articles referred to the use of gamification or “serious games”, but not all reported how theories and scientific findings supported the design choices they made for their game-based intervention. Strikingly, some articles (15.4%) did not establish any theoretical motivation at all. In those cases, the researchers' choices about the game mechanics, duration, and delivery format of the intervention were theoretically decontextualized.

We acknowledge that our review is limited in some ways: we sourced a small sample of articles, all published within the last 10 years. Certain facets of the theoretical motivation and methodological approach coded were deemed most relevant to evaluating the development and effectiveness of the game-based interventions, based on prior criticisms of the field and recommendations. However, other aspects of the reporting could still be valuable to assess. For example, the appropriateness and robustness of the statistical analyses used or the trustworthiness and rigor of qualitative analyses was not considered. Such consideration would be important for evaluating the authors' reported claims about the effectiveness of the game-based interventions, which was not the primary focus of our review. It also may have been beneficial to look at individual mechanic terms, rather than grouping them into categories. Evaluating the number of listed mechanics by authors may have aided us in answering the question of the optimal number of mechanics to include, which has been a question in past reviews (3). However, due to the variability in mechanic language, we deemed the evaluation of broad categories to be the most appropriate focus of the current review.

### 4.4 Future directions

The findings described above demonstrate that the area of research in the development and evaluation of game-based health interventions has improved in their methodologies and theory use. Our findings also demonstrate similarities across articles, despite this area of research straddling different fields. In general, studies employed large sample sizes and deployed interventions in real-world settings, such as at home or on mobile devices. While a primary driver of applied game design is improving the dissemination of interventions outside of the lab by allowing individual access through personal technology, we do recognize that this method does not reach individuals that are not able to afford smartphones or do not have access to this technology. The majority of studies also reported using comparison or control groups, permitting statistical inference about the effectiveness of the game-based intervention. As a whole, game-based interventions utilized at least two types of game mechanics and reported to have positive effects on health outcomes. Still, researchers in this area need to continue taking steps towards improving the robustness and transparent reporting of their work.

#### 4.4.1 Recommendations for development

Based on our results and our assessment of the literature, we urge researchers to guide their design choices and choice of mechanics by theory and considerations of user context. Grounding interventions in theory provides reasoning for development choices and justifies using an Applied Game Design approach. When design choices (e.g., the game mechanics used) are not backed by psychological theory or design principles, they are seemingly arbitrary, which compromises the evaluation of the intervention and its subsequent reuse.

Toward that end, echoing Khaleghi and colleagues (14) and Lukas & Palva (7), we recommend use of interdisciplinary teams comprising professionals from different fields, including experts in game development or human-centered design, as well as representatives from the groups targeted by the intervention. Interdisciplinary teams bring in a range of experience and theoretical perspectives, which can facilitate the design and implementation of game-based interventions that are attuned to the complex nature of human behavior. Such teams can facilitate the integration of multiple theoretical perspectives into the development process. Indeed, as we found, the majority of the articles that reported using interdisciplinary teams drew from both psychological and design theories. Theories from different fields each provide key insights, not only in how to motivate behavior change, but also in how to maintain change and design easy to use and engaging tasks. In interdisciplinary teams, it is feasible to make and implement choices guided by design principles and backed by sufficient technical understanding, since members have expertise in different areas, including technical ability. Such technical expertise is also helpful for handling accessibility and technical issues, as this was a barrier noted in prior criticisms. In summary, developing a game with proper theoretical grounding and with experts in game design allows effective targeting of the audience and health outcome, while still creating an enjoyable experience [see also (7, 14)].

We also recommend that research teams utilize multistage, iterative processes that utilize user perspectives and use both qualitative and quantitative evaluation methods at each stage. This design process would ensure that feasibility, usability, and efficacy are being tested and that efficient changes are made throughout the process. In terms of the process of intervention development, user-centered approaches to design, pilot, and feasibility testing are especially important, as they permit testing the effect of interventions and obtaining user feedback. This recommendation is consistent with Khaleghi and colleagues' (14) call to use prototyping and involve users throughout the development stages. These iterative, user-centered approaches confer a rich understanding of how the intervention addresses user needs and how changes to the design impact the intervention's efficacy.

#### 4.4.2 Recommendations for reporting and evaluation

As we and others have noted, the reporting of the development of game-based interventions varies greatly, which highlights the need for more consistent and transparent reporting. As stated in other reviews, a unified terminology to refer to game mechanics and development in health settings is needed (1, 24, 26). Studies either used varied terms to explicitly state which game mechanics they incorporated into their studies or did not explicitly identify those mechanics, requiring us to infer what they were based on the intervention's descriptions. The terms used in our coding scheme for game mechanics and methods can serve as the basis for a more unified way of describing game-based intervention development.

Game development and game-based approaches to intervention development are often recommended to be iterative, cyclical processes, and reporting standards should reflect this (14). Unfortunately, the reporting of the methodology in many of the reviewed studies did not reflect this ideal. It was often unclear whether processes from the predevelopment stage had already occurred or whether there were plans to incorporate feedback from the current study. Future studies in later stages of development should provide clear statements on the prior work that has been done and explain any processes they have in place to make changes to their intervention based on user feedback or other factors.

Our recommendations in response to these gaps in reporting echo those of the StaRI (Standards for Reporting Implementation Studies) initiative (35). The guidelines of the StaRI initiative focus on two primary reporting goals: describing the implementation strategy and describing the intervention and its impact. This initiative is aimed at improving intervention adoption and sustainability in healthcare, which is vital especially given recent research demonstrating the negative views of clinicians on these types of interventions (35, 36). The first goal of StaRI is for researchers to report scientific underpinnings, such as theory, that provide a rationale for the intervention and the steps of design. This recommendation resonates with our call for researchers to explicitly ground their intervention in theory and be more transparent about the steps taken during predevelopment phase. For the second goal, researchers are asked to describe intervention components (such as the game mechanics utilized) and how the intervention and its targeted outcomes were evaluated. This call aligns with our recommendation for more transparent reporting of the development and the evaluation of the game-based intervention. It may begin to address clinicians' opinion of game-based interventions and close the gap between fields, in terms of development and reporting (7, 35, 36). In fact, the authors of StaRI propose that these standards extend to different fields and evolve to accommodate different research needs (35). Given the flexibility of the StaRI standards to be adapted to the specific needs of different fields, we recommend that researchers developing game-based interventions refer to them as a resource and model their research process and reporting after them.

## 5 Conclusion

This review has highlighted key improvements, as well as continued areas of growth, in research on game-based health intervention development. It calls for more transparent reporting of methods and design choices, more intensive evaluation methods, theoretical grounding of design choices, iterative design, interdisciplinary teams, and consistent use of terminology. These recommendations, along with those from other researchers (14), aim to improve methodological rigor and facilitate the evaluation of the utility of game-based interventions. Prior mixed results on the effectiveness of game-based health interventions, along with the opaque reporting of the methods, can create hesitancy for researchers interested in adopting this approach (4, 16, 23, 24). Researchers should continue revisiting the best practices for evaluating research on game-based health interventions, as game-based applications become more common and as advances in the delivery of mobile phone interventions, as well as computer-based, VR, and AR become more pervasive.

## Data Availability

The datasets presented in this study can be found in online repositories. The names of the repository/repositories and accession number(s) can be found in the article/Supplementary Material.
