# Supplementary material for: Advancements and challenges in methodological approaches for game-based health interventions: a scoping review
Source: Front Digit Health. 2025 Mar 24;7:1561422. doi: 10.3389/fdgth.2025.1561422 (PMC11973360; doi:10.3389/fdgth.2025.1561422)
Supplement: Supplementary file 1 [file Datasheet1.docx]

**Theory Coding Manual**

**Theories were designated into 1 of 2 categories:**

- Game Design/Development Theories
- Psychological Theories

**Definitions**

- Game Design/Development Theories: theories used for guiding a choice in the design of the game
- Psychological Theories: theories used as support for how aspects of the game impact human thought and behavior

**Examples**

- Game Design/Development Theories: flow theory, goal setting theory, MEEGA+, narrative transportation theory, user-centered design, input-process-output model, four-dimensional framework, game-based evaluation model, the proteus effect, etc.
- Psychological Theories: social-determination theory, social cognitive theory, indivisible self model, wellness theory, protection motivation theory, social incentive theory, behavioral economics, prospect theory, etc.
