# Supplementary material for: Advancements and challenges in methodological approaches for game-based health interventions: a scoping review
Source: Front Digit Health. 2025 Mar 24;7:1561422. doi: 10.3389/fdgth.2025.1561422 (PMC11973360; doi:10.3389/fdgth.2025.1561422)
Supplement: Supplementary file 3 [file Datasheet3.docx]

**Methods Coding Manual**

**Step 1: Identified Unique Terms from the literature**

feasibility testing/beta testing/pilot testing/mock up

Interviews

Focus groups (qualitative)

Multistage

RCT

Interdisciplinary team

Mixed methods approach

user feedback/participant input

prototype

iterative process

activity based planning

evaluation study

co-creation

surveys/questionnaire

experiment

video recordings

acceptability

credibility

past validation

longitudinal

bio measures

patient-centered

cluster randomized trial

qualitative survey

formative process

thematic analysis

**Possible Areas for Distinction**

From Khaleghi et al. (2021) paper:

Pre-development - knowing users, prototyping

development - testing w/ users; getting feedback

post development - implementation

Iterative/cycle

Interdisciplinary

**Research Design Types in the literature**

Qualitative

Quantitative

Experimental

**Attempt 1 at coding methods:**

We initially attempted to divide methods by where they may be used in Khaleghi and colleagues' (2021) stages

pre-development

Feasibility, prototype, acceptability, credibility, past validation

Development

Quantitative - surveys, questionnaires, experiments, RCT, cluster randomized trials, bio measures

Qualitative - interviews, focus groups, qualitative survey, video recordings

Mixed Methods (both quantitative and qualitative)

Post development *we had trouble designating terms to this stage

Terms we originally had uncertainty about:

activity based planning (Qual?)

evaluation study (Quant?)

user feedback/participant input (Can be pre / dev / post-development; can be Quant or Qual?

*However, we determined this was an inadequate way to categorize methodologies as methods can span across developmental stages, and it was unclear what would specifically go under post-development. There was also a set of terms that we did not know how to group within the above categorization.

**Final Categorization**

We decided we wanted to determine:

- The stage of development studies were in
- Whether studies use qualitative methods, quantitative methods, or both
- Whether studies used user-centered approaches
- Whether studies reported engaging in testing their interventions before implementation
- Whether studies discussed a process for integrating user feedback into the development of their interventions, in other words, an iterative design process
- If studies indicated the use of an interdisciplinary team

Categorization terms:

- Stage (pre-development, development, post development)
  - This category specified which stage the paper seems to be in (requires a personal judgment)
    - Judgements were based on the definition of stages
      - Predevelopment - if prototyping and feasibility testing was conducted, an indication of still being in the planning phase, or stated their goal was to improve the initial development of the intervention
      - Development - if they were testing the efficacy of their intervention
      - Post-development - if the researchers stated their intervention had already been determined to be effective, stated their goal was to test its efficacy in other populations (other than what it was originally developed for), or if the study involved disseminating/applying the intervention.
- Mixed methods (yes/no)
  - if mention both quantitative and qualitative methods
- Quantitative:
  - Examples: surveys/questionnaires, experiment, RCT, cluster randomized trials, biological measures (BMI, steps, etc)
- Qualitative:
  - Examples: interviews, focus groups, qualitative surveys, video recordings, thematic analysis
- User-centered approach:
  - Examples: user feedback/participant input, co-creation, patient-centered
- Testing:
  - Examples: feasibility testing/beta testing/pilot testing/mock-up, prototype, acceptability, credibility, past validation, evaluation study (described as testing efficacy)
- Feedback integration
  - Examples: multistage, iterative, activity-based planning (involves observing how the tool is used to collect feedback and make updates), formative process
- Interdisciplinary (yes/no)
  - The paper explicitly mentioned the use of interdisciplinary teams or a mix of experts and/or end-users within the research team

*This categorization leaves out specifications about longitudinal or cross-sectional studies, but we had a section on the duration of intervention already, so we decided to keep this separate
