# Supplementary material for: Advancements and challenges in methodological approaches for game-based health interventions: a scoping review
Source: Front Digit Health. 2025 Mar 24;7:1561422. doi: 10.3389/fdgth.2025.1561422 (PMC11973360; doi:10.3389/fdgth.2025.1561422)
Supplement: Supplementary file 4 [file Datasheet4.docx]

**Mechanic Coding Manual**

**Step 1: Unique Terms from the Literature *synonymous terms are on the same line**

weekly challenges, challenges;

self progression

game visuals; artwork; three dimensional environments; virtual environments

Free roam of env

Self-representation with avatars;

social message network,

Parallel communication; AI communication

rewards: point system (each step recorded and food logs); rewards (points), Digital rewards (points); badges,

punishment: removal of points, levels

rewards: badges; Digital rewards (points, badges); badges,

rewards: Real-world prizes;

rewards: level up

narrative, Narrative context (or story);

timed events, Time pressure, time limit

goal setting, goals

progress tracker; progress feedback; progress visualization

feedback, immediate feedback

teams,

Competitions

Leaderboards

ranks

Social or peer pressure;

Practice, dosing

Levels

Marketplaces and economies

Rules

Randomness/suprises

**Step 2: Color Coded Related Terms *black text indicates uncertainty in how to categorize**

weekly challenges, challenges;

self progression

game visuals; artwork; three dimensional environments; virtual environments

Free roam of env

Self-representation with avatars;

social message network,

Parallel communication; AI communication

rewards: point system (each step recorded and food logs); rewards (points), Digital rewards (points); badges,

punishment: removal of points, levels

rewards: badges; Digital rewards (points, badges); badges,

rewards: Real-world prizes;

rewards: level up

narrative, Narrative context (or story);

timed events, Time pressure, time limit

goal setting, goals

progress tracker; progress feedback; progress visualization

feedback, immediate feedback

teams,

Competitions

Leaderboards

ranks

Social or peer pressure;

Practice, dosing

Levels

Marketplaces and economies

Rules

Randomness/suprises

**Possible Categorizations:**

reinforcement/scoring (encompasses rewards and punishment)

Immersion (story/narrative, environement, exploration)

Status (competition, leaderboard, social/peer pressure, collaboration)

Goals (goal setting, assigned challenges, levels, self-progression?)

Progress (tracking, feedback?)

*we were unsure how to categorize feedback as we can see it either being used to inform players of their progress or to be encouraging and act as a motivator

Feedback (as a separate category; maybe different types of feedback will emerge: about accuracy, about progress, etc)

Collaboration/interaction: social messaging, AI communication, marketplaces and economies (maybe under reinforcement depending on context)

time manipulations: timed events, pressure, limit, dosing, randomness/surprises

**Transition to Final:**

To finalize our categories, we referenced past literature:

- Hervás, R., Ruiz-Carrasco, D., Mondéjar, T., & Bravo, J. (2017, May). Gamification mechanics for behavioral change: a systematic review and proposed taxonomy. In Proceedings of the 11th EAI International Conference on Pervasive Computing Technologies for Healthcare (pp. 395-404). <https://dl.acm.org/doi/pdf/10.1145/3154862.3154939>
- Green, M. C., Khalifa, A., Canaan, R., Bontrager, P., & Togelius, J. (2021, August). Game Mechanic Alignment Theory. In Proceedings of the 16th International Conference on the Foundations of Digital Games (pp. 1-11). <https://arxiv.org/pdf/2102.10247.pdf>
- Toda, A. M., Klock, A. C., Oliveira, W., Palomino, P. T., Rodrigues, L., Shi, L., ... & Cristea, A. I. (2019). Analysing gamification elements in educational environments using an existing Gamification taxonomy. Smart Learning Environments, 6(1), 1-14. <https://link.springer.com/article/10.1186/s40561-019-0106-1>

We also discussed whether terms can be merged and the purpose of the mechanics in gameplay.

**Final Categorization:**

- Reinforcement
  - Examples: Rewards (points, badges, medals, real-world rewards), punishment (point reduction), feedback
- Immersion
  - Examples: Story/narrative, virtual or physical environment, ability to explore the environment
- Performance
  - Examples: Goal setting, assigned challenges, levels, progress tracking
- Social
  - Examples: Competition, leaderboard, social/peer pressure, collaboration, social messaging, AI communication (intelligent communication, not typical NPC interactions)
- Ecological
  - Examples: Timed events/limits, timed pressure, dosing, randomness/surprises, marketplace/economies
